# Posterior Fixation Techniques in the Subaxial Cervical Spine

**DOI:** 10.7759/cureus.338

**Published:** 2015-10-01

**Authors:** Ahmer Ghori, Hai V Le, Heeren Makanji, Thomas Cha

**Affiliations:** 1 Orthopedic Surgery, Massachusetts General Hospital; 2 Orthopaedics, Brigham and Women's Hospital

**Keywords:** spine, subaxial spine, posterior fixation, cervical spine, pedicle screw, lateral mass, laminar screw

## Abstract

This article reviews the historical context, indications, techniques, and complications of four posterior fixation techniques to stabilize the subaxial cervical spine. Specifically, posterior wiring, laminar screw fixation, lateral mass fixation, and pedicle screw fixation are among the common methods of operative fixation of the subaxial cervical spine. While wiring and laminar screw fixation are now rarely used, both lateral mass and pedicle screw fixation are technically challenging and present the risk of significant complications if performed incorrectly. With a sound understanding of anatomy and rigorous preoperative evaluation of bony structures, both lateral mass and pedicle screw fixation provide a safe and reliable method for subaxial cervical spine fixation.

## Introduction and background

Posterior fixation of the subaxial spine is routinely performed for cervical spinal instability from any etiology, such as trauma, infection, primary or metastatic malignancy, or decompressive laminectomy. It is often done to augment subaxial spine stabilization in conjunction with multilevel anterior decompression fusion procedures. Multiple posterior approaches for surgical fixation of the subaxial spine have been developed and continually modified over the years with a better understanding of subaxial spine anatomy and biomechanics, concurrent with improved spine implant technology. This article reviews four common fixation techniques, which comprise posterior cervical wiring, laminar screw fixation, lateral mass screw fixation, and pedicle screw fixation. With each technique, we detail its historical context, indications, contraindications, techniques, and recommendations for complication avoidance.

## Review

### Wiring

Introduction

Posterior cervical wiring historically has played a major role in stabilizing the cervical spine. Hadra first introduced spinous process wiring in Pott’s disease in 1891 [1], and Rogers described in detail techniques for interspinous wiring of the cervical spine in 1942 [2-3]. Rogers’ technique was later developed and modified by Abdu and Bohlman (triple-wiring) [4], Whitehill, et al. [5], Benzel and Kesterson [6], and Murphy and Southwick [7]. Although wiring restores the posterior tension band construct, it does not stabilize against extension, rotation, or lateral bending. Since the introduction of fusion and instrumentation with plates and screws, posterior cervical wiring today has a very limited role in fixation of the subaxial spine, usually functioning as a salvage procedure or as an adjunct to other fixation constructs [8].

Indications

Posterior cervical wiring requires that the posterior bony ring is preserved (i.e., lamina, facet, or spinous process). Sublaminar wiring has been shown to be cost-effective with low neurological risks in patients with cervical spine trauma [9-10]. Because posterior tension banding stabilizes flexion motion, it can be utilized in a flexion-distraction injury with facet subluxation or dislocation [11]. It can also be used to augment anterior cervical instrumentation. Finally, stand-alone wiring is generally limited to pathology involving just one cervical level [12].

Contraindications

Any traumatic or pathologic process that compromises the integrity of the posterior bony elements (i.e., lamina, facet, or spinous process) is a contraindication for posterior cervical wiring. In addition, wiring only offers resistance against flexion, so additional fixation technique(s) must be employed to provide extension, rotation, and lateral bending stability. Tension band wiring is at risk for failing in osteoporotic bone. In general, stand-alone posterior fixation is relatively contraindicated when there is instability of the anterior or middle column.  

Techniques

There are many posterior wire stabilization techniques that have been developed and described in the literature. We will review the original Rogers’ interspinous wiring technique and one of its modifications, Abdu's triple-wiring technique, which has been shown to impart greater biomechanical stability [13].

**Figure 1:**
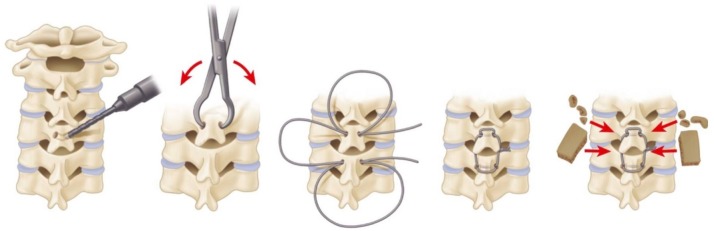
Rogers Interspinous Wiring Technique A burr hole is drilled transversely at the base of the upper and lower spinous processes. A stainless steel or titanium wire or cable is passed through the burr holes in a figure eight pattern and subsequently tightened.

In the Rogers’ interspinous technique, a burr hole is drilled transversely at the base of the upper spinous process and the lower spinous process. A stainless steel or titanium wire or cable is passed through the burr holes in a figure eight pattern. Finally, the wire is tightened using a tensioner (Figure 1).

**Figure 2:**
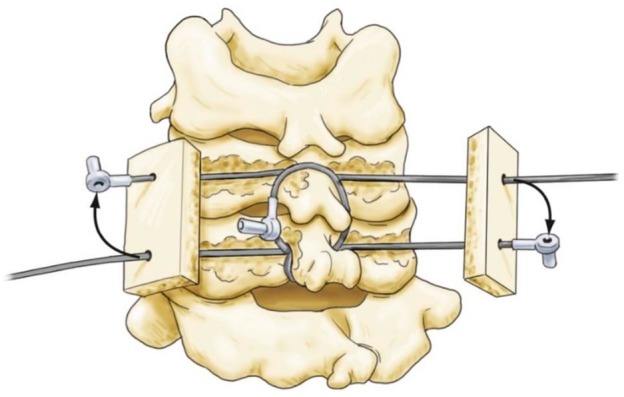
Bohlman Interspinous Wiring Technique The first wire is secured in similar fashion to the Rogers technique. Second and third wires are then passed through the upper and lower burr holes and looped around the upper and lower spinous processes, respectively. Finally, the latter two wires are secured to autologous bone graft struts and tightened.

With Abdu’s triple-wiring, the wire is first secured under tension as described by the Rogers’ technique. Subsequently, the second wire is passed through the upper burr hole and looped around the upper spinous process. Similarly, the third wire is passed through the lower burr hole and looped around the lower spinous process. These latter two wires are then passed through corresponding holes drilled in two autologous bone graft struts, each placed lateral to the spinous processes. These wires are then tightened under tension (Figure 2) [13-14].

Complications

The main complications from posterior cervical wiring are wire pullout and injury to the spinal cord or exiting spinal nerves from the misdirection of the wire. Fortunately, these complications are rare [10]. Over-tightening of the wire in facet wiring can lead to avulsion fractures. Sublaminar wiring is almost never used in the subaxial spine because the spinal canal is smaller compared to the spinal canal at the C1/C2 levels. This becomes more problematic in patients with degenerative or congenital cervical stenosis. Loss of fixation from poor bone quality or inadequate postoperative immobilization are other complications. Finally, as with other fixation techniques, nonunion, malunion, or hardware infection can be observed with posterior cervical wiring [15].

Complication Avoidance

As with any surgery, comprehensive preoperative planning is necessary to study the anatomy and evaluate for anatomical variations in order to plan the operation accordingly. Patients with anterior or middle column instability will need additional anterior fusion and instrumentation. Sublaminar wiring should be avoided in patients with a narrowed spinal canal or neural foramen. Avoid kinking during wire passage to prevent creating a stress riser where wire breakage can occur. The tension should be sequentially tightened with the tensioner as to avoid loosening as well as over-tightening. When drilling at the base of the spinous process, avoid drilling too deeply, and angle the drill transversely (horizontally) to avoid violating the epidural space.   

### Laminar screw fixation

Introduction

Traditionally, translaminar screw fixation is better described and more frequently performed in the atlantoaxial and thoracolumbar spine [16-17]. Laminar screw fixation in the subaxial spine remains an uncommon practice and is utilized in only selected cases. As such, the literature is very limited on this topic. In two recently published simulation studies evaluating the feasibility of translaminar screw placement in the subaxial spine, the authors concluded that C7 had a high unilateral screw placement success rate (100% for 3.5 mm screw, 91.7% for 4.0 mm screw) and moderate bilateral screw placement success rate (90% for 3.5 mm, 68.8% for 4.0 mm). At C3-C6, the success rates were much lower [18-19]. The feasibility of translaminar screw placement at C7 is primarily due to its larger laminar size. Clinically, laminar screw fixation has had promising results with low complication rates; the main complication reported was dorsal laminar breach [20-21]. However, further studies with larger subject populations are needed to evaluate the clinical feasibility of translaminar screw fixation in the subaxial spine.

Indications

Laminar screws may be used in cases of deficient lateral masses or after failed attempts to place a lateral mass screw. They appear to have better biomechanics at C7 when compared to the C3-C6 levels. As with any posterior fixation technique, translaminar screw fixation requires intact posterior elements, specifically intact laminae, at the levels to be instrumented. 

Contraindications

Any traumatic or pathologic process that leads to insufficiency of the lamina is a contraindication to laminar screw fixation (e.g., post-laminectomy deformity). As with any posterior fixation technique, stand-alone translaminar screw fixation is insufficient to restore stability in cases involving the anterior and/or middle columns. 

Techniques

**Figure 3:**
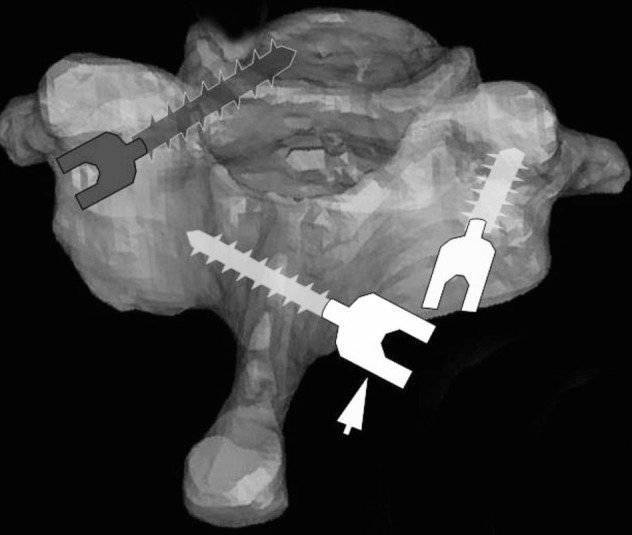
Translaminar Screw Fixation The arrow is pointing to theoretical position of a translaminar screw from one spinolaminar junction to the opposite laminar-lateral mass junction. Compare to position of lateral mass screw (right) and pedicle screw (left).

We will review the technique for C7 screw placement as described by Hong, et al. and Jang, et al. in 2008 and 2010 (Figure 3) [20-22].

Preoperative axial CT imaging is obtained to measure the translaminar screw length as well as trajectory angle and path. The screw length is estimated as the length from the contralateral spinolaminar junction to the ipsilateral lamina/lateral mass junction [20]. An entry point is created with a high-speed drill at the spinolaminar junction. The trajectory is created by using a hand drill and proceeding to the estimated depth. Screw length (usually between 20-30 mm) is checked with a depth gauge, making sure that the laminar cortical bone has not been violated. Finally, a 3.5 or 4.0 mm screw of proper length is fastened and secured. If laminar screws are placed bilaterally, the entry points for the screws are staggered at the base of the spinous process (spinolaminar junction) so the two screws do not collide during insertion [22].

Complications

The complications of laminar screw fixation include laminar cortical breach and violation of the facet joint. Breach of the medial cortex can lead to spinal cord injury and penetration of the thecal sac. Screw loosening and hardware failure are other reported complications [20-22].

Complication Avoidance

Although injury to the vertebral artery is theoretically lower with intralaminar screw placement, a long drill or screw can potentially injure the lateral structures, including the vertebral artery. Preoperative CT is warranted to measure the size of the lamina and estimate the length of the screw to ensure there is adequate bone stock and laminar diameter for good screw purchase. Intraoperative fluoroscopic imaging can assist in identifying the ideal trajectory for screw placement to prevent penetration of the cortical walls.

### Lateral mass screw fixation

Introduction

Lateral mass screw fixation is widely considered the mainstay technique to achieve posterior fixation of the subaxial spine. Roy-Camille first introduced posterior cervical spine fixation with lateral mass screws in 1964 [23]. This fixation technique was subsequently popularized by Louis [24] and Magerl [25-26] and more recently by Anderson [27] and Ebraheim [28]. Anatomically, the lateral mass, or articular mass, consists of the superior and inferior articular facets. The lateral mass lies anterolateral to the lamina. Posterior stabilization utilizing the lateral mass offers exceptionally high fusion rates, with ranges between 85-100% reported in the literature [24-29].

Indications

Lateral mass screw fixation provides strong posterior fixation in patients with instability of the subaxial spine. Lateral mass screws have been implemented with success in cervical spine trauma, infections (i.e., osteomyelitis), neoplasms, degeneration, and failed anterior fusions. Lateral mass screw construct offers comparable or superior stability compared to pedicle screw fixation or laminar screw/sublaminar wiring and is a useful surgical option in patients whose pedicles or laminae are deficient [28, 30].     

Contraindications

Since lateral screw fixation requires an intact lateral mass, any traumatic or pathologic process that compromises the integrity of the lateral mass is a contraindication for this type of fixation. In cases of severe instability, lateral mass screws may not offer sufficient stabilization. As such, posterior fixation may have to be supported with anterior stabilization, thus subjecting the patient to an additional procedure [31]. In certain trauma cases, spondyloarthropathies, osteoporosis, metastatic disease, and revision surgery, the posterior elements may be comminuted or deficient enough to preclude lateral mass fixation [31-33]. The aforementioned situations highlight the limitations of lateral mass screws, and in these cases, pedicle screws can be useful.

Techniques

**Figure 4:**
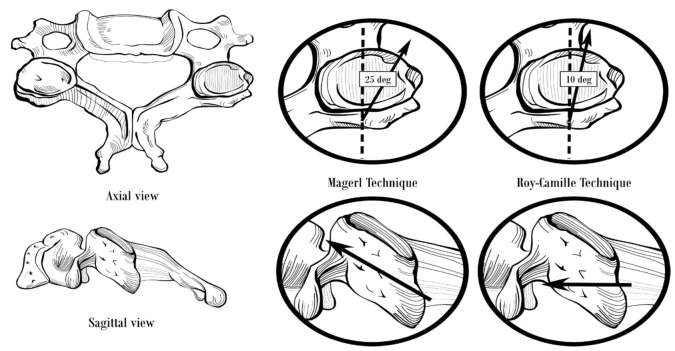
Lateral Mass Screw Fixation Starting point and trajectory of lateral mass screws placed by the Magerl and Roy-Camille techniques.

We will describe two commonly used techniques for lateral mass screw placement, the Roy-Camille’s technique and the Magerl’s technique, which differ in regards to the entry point and screw trajectory (Figure 4).

The starting point for the Roy-Camille’s technique is at the midpoint of the lateral mass. The lateral mass is rectangular-shaped when viewed from behind (posteriorly). A vertical line can be drawn connecting the facet joints at the midline, and a horizontal line can be drawn dividing the lateral mass into equal upper and lower halves. The intersection of these lines is the ideal entry point for the Roy-Camille’s technique. An entry hole is created using a 2 mm drill bit, angling perpendicular to the posterior lateral mass wall and 10 degrees lateral to the sagittal plane [23, 28, 34]. Next, the drill hole is tapped with a 3.5 mm tap, and a probe may be inserted to confirm that the lateral mass walls have not been violated. A depth gauge is inserted to measure the screw length and the appropriate size screw is inserted [28].

With the Magerl’s technique, begin by identifying the center of the lateral mass, which is the entry point for the Roy-Camille’s technique described above. The Magerl’s starting point is 1 mm medial and 1 mm cephalad in relation to the Roy-Camille’s starting point. The trajectory is angled at 45-60 degrees anterosuperiorly (parallel to the overlying facet joint) and 25 degrees lateral to the sagittal plane [28, 34]. The screw length is typically 14 mm for Roy-Camille compared to 18 mm for Magerl [34]. The Roy-Camille’s technique is reported to have a lower risk of nerve root injury while the Magerl’s technique is reported to have a lower risk of facet joint violation [35].

Complications

Neurovascular structures are at risk when inserting lateral mass screws. Misplacement of the lateral mass screws can cause injury to the spinal cord, vertebral artery (extremely rare), spinal nerves, and facet joints. Since the trajectory is directed away from the spinal cord, lateral mass screw insertion, in theory, has a lower risk of injuring the spinal cord. Neurologic injury can also be caused by insertion of long screws leading to a violation of the ventral cortex of the lateral mass. Aiming the screw anteromedially rather than anterolaterally can lead to penetration of the transverse foramen and thus put the vertebral artery at risk. Other common complications include screw loosening and pull out [36]. Although these complications are possible, lateral mass screws have an excellent safety profile: Two studies, with a combined 2,687 lateral mass screws placed, found no cases of vertebral artery, exiting nerve, or spinal cord injury that was attributable to the screw placement [37-38].

Complication Avoidance

Preoperative anterolateral (AP) and lateral plain radiographs should be obtained to identify the level(s) of interest and to understand the anatomy. Computed tomography (CT) imaging is recommended for preoperative planning. Intraoperative fluoroscopy is utilized to identify the ideal starting point and guide the trajectory during screw placement. If a patient becomes symptomatic at follow-up, plain films followed by CT imaging are recommended to determine screw loosening or failure. MRI is often required to determine whether an over-penetrated lateral mass screw has damaged the exiting spinal nerve. 

The vertebral artery lies within the transverse foramen, medial and anterior to the lateral mass. Injury of the vertebral artery is avoided by directing the screw laterally, in an "up and out" direction. Although bicortical screw purchase offers greater pullout strength compared to unicortical screw purchase, it inherently carries a greater risk of over-penetration and thus injury to nerve roots and vertebral artery [39].

### Pedicle screw fixation

Introduction

Pedicle screws are the standard of care in the thoracic and lumbar spine but are not routinely used in the cervical spine because they are technically difficult to place. This is due to small pedicle diameter and high medial angulation. Pedicles get smaller caudal to C2, reaching a nadir around C3-C4 [40], and 75% of C3-C4 pedicles have an average diameter less than 4 mm [41]. Cadaveric studies have demonstrated high rates of pedicle perforation with screw placement [42-44]. Furthermore, the lateral wall is the thinnest structure in the pedicle making screw perforation into the vertebral artery a significant risk [41]. The medial angulation of pedicles increases in the subaxial cervical spine [45]. Therefore, in order to match the pedicle trajectory, a far lateral exposure is required.  Often times, it is not possible to retract the posterior neck musculature to get adequate exposure.

Indications

Lateral mass screws are increasingly being used for posterior cervical fixation in the United States. Although they may serve well in the majority of cases, they have their limitations. Given their low pullout strength in cases of severe instability, they may not offer sufficient stabilization [45]. As such, posterior fixation may have to be supported with ventral stabilization, subjecting the patient to an additional procedure [45]. In certain trauma cases, spondyloarthropathies, osteoporosis, metastatic disease, and revision surgery, the posterior elements may be comminuted or deficient, such that lateral mass fixation is not possible [45-47]. The aforementioned situations highlight the limitations of lateral mass screws, and this is where pedicle screws can be useful.

Several biomechanical studies have demonstrated that pedicle screws offer superior fixation when compared to lateral mass screws [48-50]. Their relative pullout strengths in two studies were 1214 N vs. 332 N [48], and 677 N vs. 355 N [49]. Under cyclic loading, pedicle screws have been found to fail due to pedicle fracture rather than screw pullout [48], whereas lateral mass screws tend to loosen and pull out due to poor fixation [51]. Pedicle screws lead to consistently high rates of fusion, and this has been demonstrated across a variety of challenging scenarios: spondyloarthropathy/inflammatory arthropathy/metastatic cancer [52-53], trauma [54], cases with deficient lateral masses [55], in correcting kyphosis [56], and offering a solid construct for occipitocervical [57] or cervicothoracic fixation [58]. Thus, there is a clear role for pedicle screw fixation of the subaxial cervical spine in certain types of patients.

Techniques

**Figure 5:**
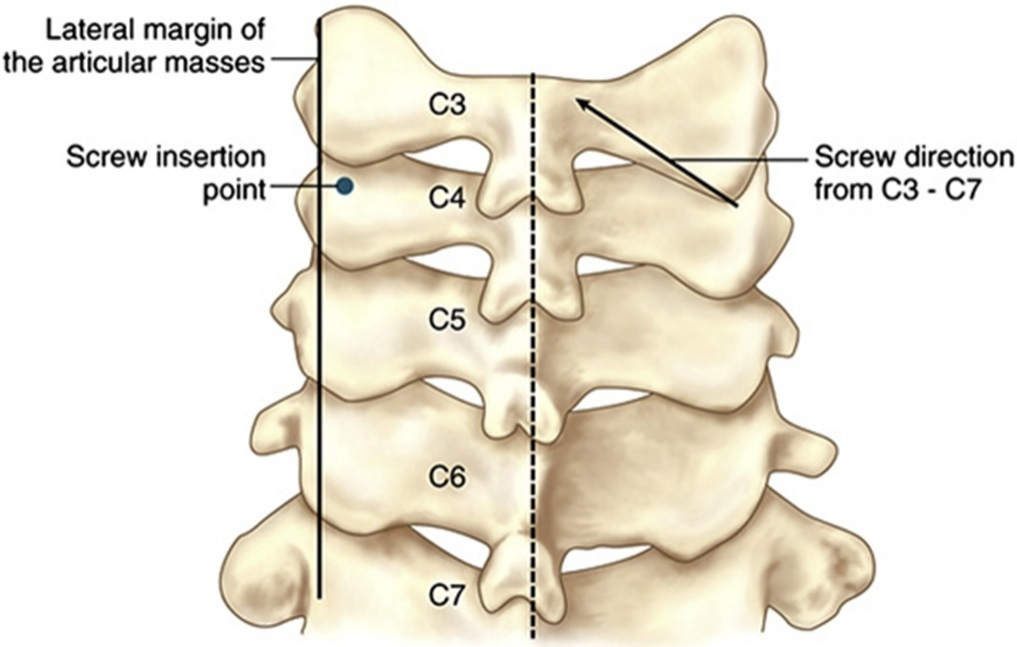
Pedicle Screw Fixation Starting point for subaxial cervical spine pedicle screw placement as described by Abumi, et al. 1994.* (Picture* *adapted from Pelton, et al. (2012) [46].)*

Abumi, et al. first described a technique for pedicle screw placement in 1994 [54]. The starting point is 1 mm lateral to the center of the articular mass, near the cranial end of the superior articular process (Figure 5).

**Figure 6:**
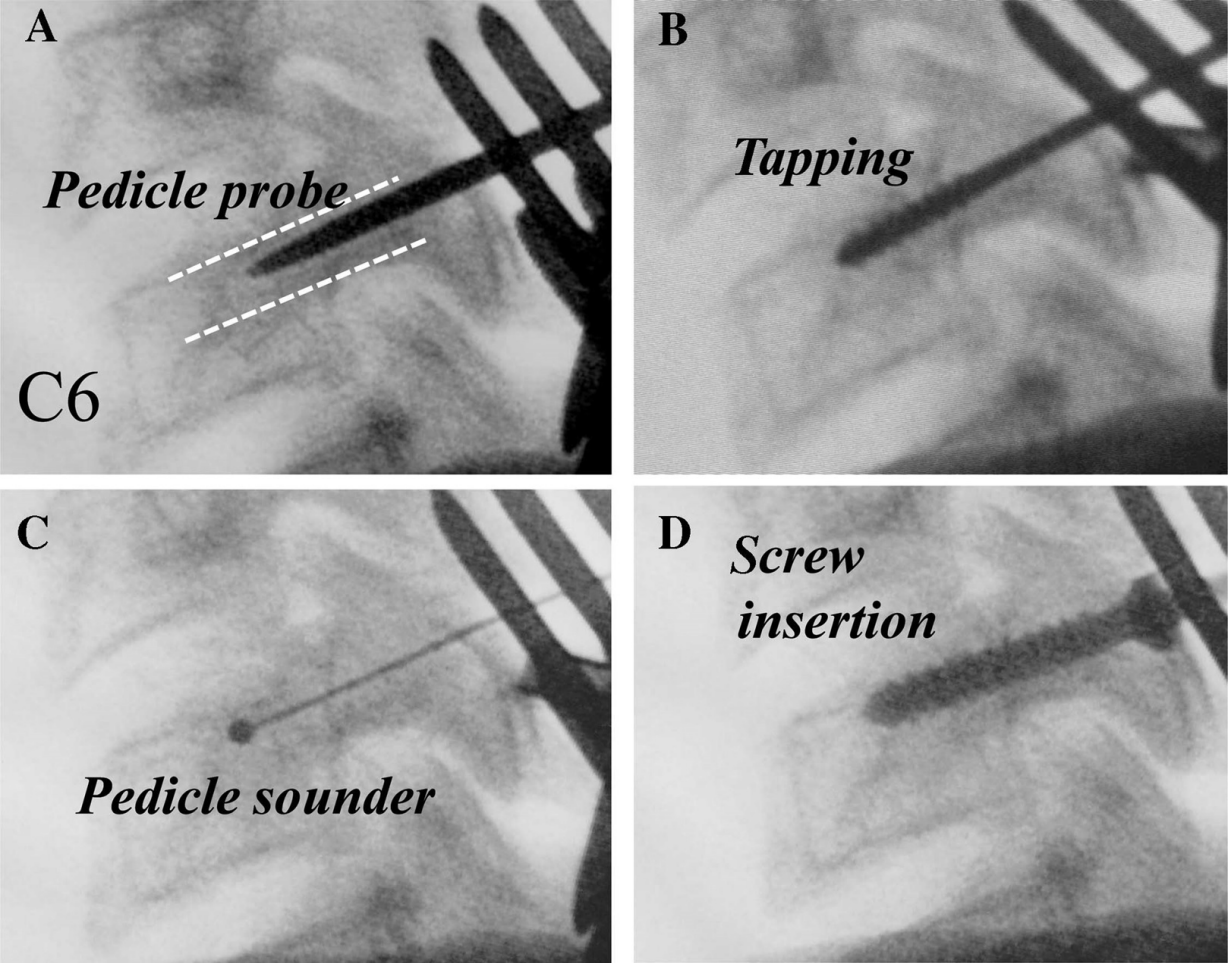
Pedicle Screw Placement Steps in subaxial cervical spine pedicle screw placement. *(Adapted from Abumi, et al. (2012) [33].)*

A high-speed burr is used to decorticate the starting point to expose the pedicle canal. A small pedicle probe is then inserted into the canal with the help of a lateral image intensifier. The pedicle is tapped under fluoroscopic guidance, and finally, an appropriately sized screw is inserted (Figure 6).

**Figure 7:**
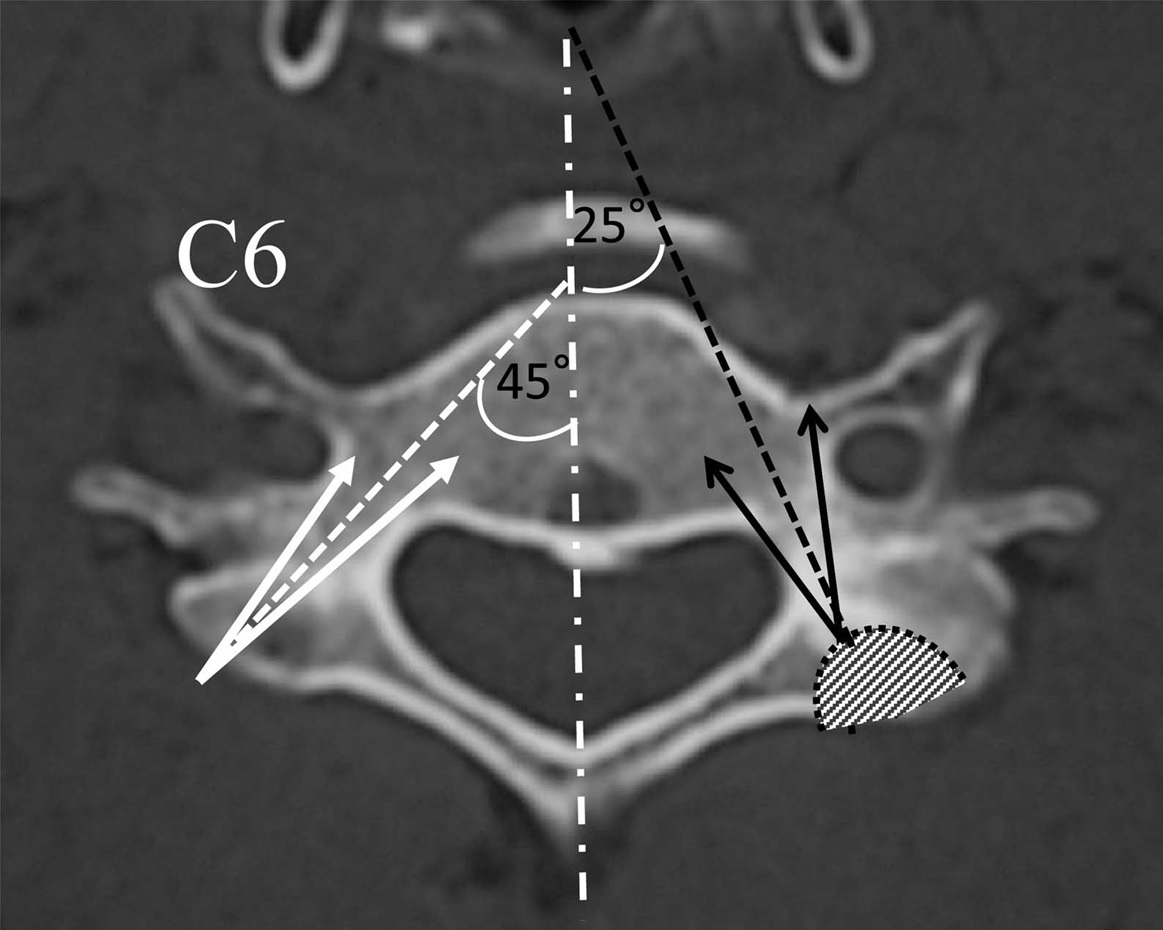
Pedicle Screw Angulation Medial to lateral angulation of subaxial cervical spine pedicles. *(Adapted from Abumi, et al. (2012) [33].)*

While placing pedicle screws, it is important to consider the location of the pedicle in three-dimensions. The medial to lateral pedicle angulation is variable and often determined from preoperative imaging. In general, this angle is lowest at C2 and increases caudally. Abumi, et al stated that most of their screws ranged from 25 to 45 degrees from the transverse process in the horizontal plane [47]. Figure 7 demonstrates the horizontal plane trajectory for pedicle screw fixation in the subaxial cervical spine.

The cranial-caudal angulation of pedicles is superior to the vertebral endplate at C2-C3, parallel to the end plate at C3-C4, and inferior to the endplate at C5-C6 [41].

Since Abumi’s initial description in 1994, several different techniques have been described. They vary in how the starting point is obtained, and options include using surface landmarks, performing a laminoforaminotomy to probe the pedicle borders, and using computer navigation. The relative merits of these techniques are discussed later in the chapter.

Complications

**Figure 8:**
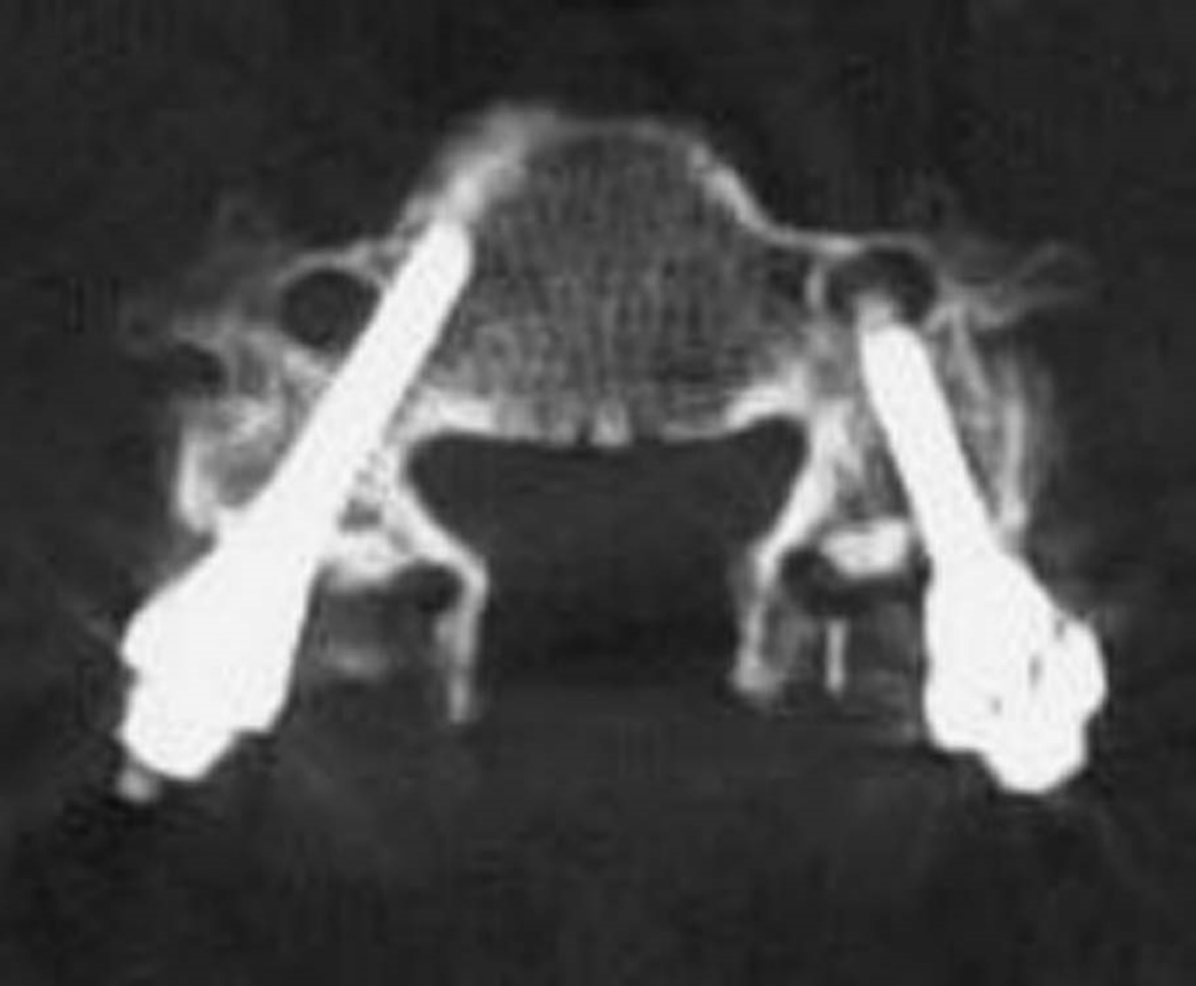
Lateral Pedicle Wall Breach Lateral pedicle wall breach with transverse foramen penetration.

Given the small pedicle diameter, high medial to lateral angulation, and thin lateral cortex, this is a technically demanding procedure. Complications can be broadly categorized into injury to the vertebral artery, spinal cord, or exiting nerves. A lateral pedicle perforation would lead to violation of the transverse foramen with potential vertebral artery injury (Figure 8).

**Figure 9:**
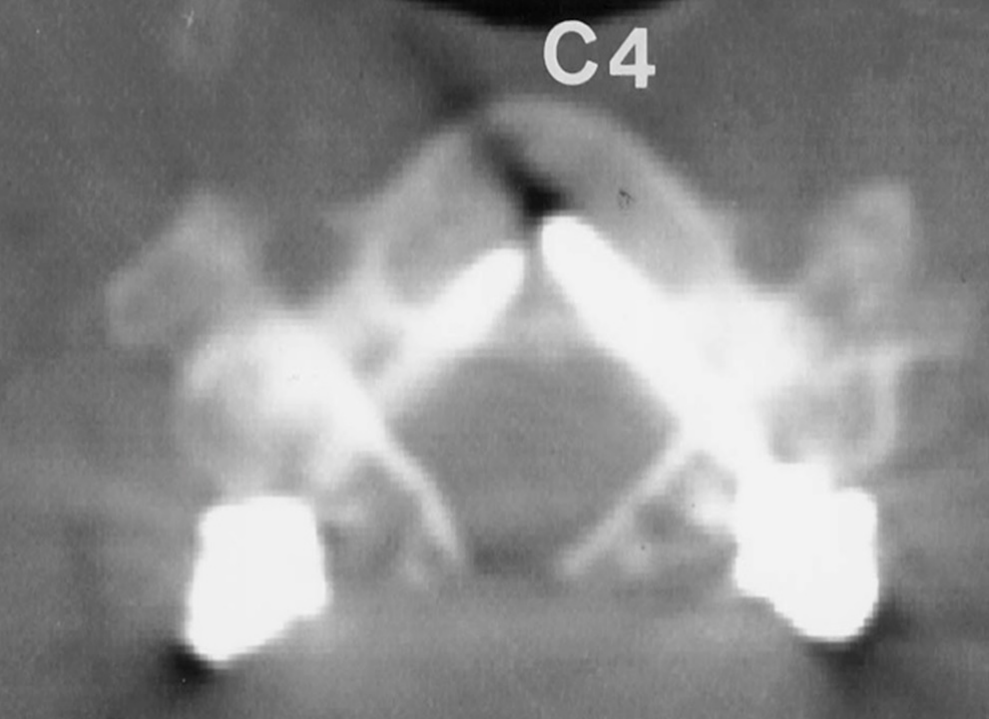
Medial Pedicle Wall Breach Medial pedicle wall breach with spinal canal violation.

A medial perforation would violate the spinal canal and risk dural tear or spinal cord injury (Figure 9).

A superior or inferior breach would violate the neural foramen and could cause nerve injury.

Five studies in the literature have analyzed complications from pedicle screw fixation in the subaxial cervical spine. The rates of screw perforation ranged from 6.7% to 30%, and most cases breached the lateral wall [45, 59-62]. The most common risk factor for screw malposition was level of surgery: in one study, 91% of the screws at C6 were correctly placed, compared to only 48% of screws at C4 [45]. This finding was explained by the small pedicle size and high horizontal angulation of pedicles from C3-C5.

Despite the relatively high rate of pedicle perforation with screw placement, the incidence of neurovascular injury is relatively low. Out of the 350 patients across the five studies, only two patients had a vertebral artery injury, five patients had a nerve root injury, and zero patients had a spinal cord injury [45, 59-62]. None of the cases of vertebral artery injury led to cerebral ischemia or other neurologic deficits. Most cases of nerve injury led to temporary neurologic deficits, which resolved over time with conservative management. Only a minority of cases with nerve injury required pedicle screw revision.

This high incidence of pedicle perforation, yet the low incidence of neurovascular injury, can be explained on an anatomic basis. On average, the vertebral artery occupies only 35% of its foramen. Furthermore, the distance from the vertebral artery to the lateral pedicle wall increases from C2 to C7 [63]. The critical amount of pedicle breach that would predict vertebral artery injury has yet to be determined. In the cervical spine, nerves occupy the inferior half of the neural foramen, and they exit at 45 degrees to the coronal plane and 10 degrees sagittal plane [64-65]. Exiting nerves lie nearly opposed to the superior part of the caudal pedicle and lie 1.1–1.7 mm from the inferior aspect of the cranial pedicle [65]. Therefore, a superiorly placed pedicle screw is more likely to cause nerve damage compared to an inferiorly placed screw. The medial wall of the pedicle is thickest and the dural sac is 2.4–3.1 mm away, which may explain why there have been no reported cases of spinal cord injury from subaxial cervical pedicle screw placement [63].

Complication Avoidance: Learning Curve

Subaxial cervical pedicle screw placement is not a commonly used technique and is technically demanding. As such, there is a learning curve and published results demonstrate improved outcomes with surgeon experience. In one study, screw misplacement was 13% for the first 20 screws and decreased to 4% for the subsequent screws [64]. In another study, all of the complete pedicle perforations occurred in the first 10 patients, with no perforations in the subsequent patients [60]. Therefore, safe subaxial cervical pedicle screw placement requires instruction and appropriate supervision from experienced surgeons.

Complication Avoidance: Computer Navigation

As described earlier, the starting point can be obtained based on a number of methods. As expected, the literature shows improved accuracy with more detailed delineation of pedicle anatomy. One study reported a 65% pedicle breach with using surface landmarks alone, 39.5% with laminoforaminotomy, and 10.5% with computer navigation [44]. Another study reported an 8.6% pedicle perforation with surface landmarks and 3% with navigation [66]. Yet another study showed a perforation rate of 6.7% with conventional technique and 1.2% with a surgical navigation system [67]. Overall, the literature suggests the most accurate pedicle screw placement with computer-assisted navigation. However, previous studies have shown pedicle perforation does not necessarily translate into neurovascular injury [68]. Therefore, it is unclear if the added expense of computer-assisted navigation would be cost-effective in the long term.

Complication Avoidance: Imaging

The main complications with pedicle screw placement - pedicle breach, vertebral artery injury, and nerve injury - can be minimized with appropriate preoperative imaging. Pedicle diameters are small in the subaxial cervical spine and tend to vary across the population. In general, pedicle screws are unsafe when pedicle diameters are less than 4 mm. Therefore, preoperative CT scan should be obtained to make sure pedicles are large enough to be instrumented. Vertebral artery occupancy in the transverse foramen can be obtained with preoperative CTA. There have been described cases where CTA reveals a  tortuous artery with a high occupancy or one that erodes into the pedicle wall. In such cases, pedicle screws should be avoided. In general, a sound understanding of the pedicle anatomy and its associated neurovascular structures for individual patients will help reduce complications.

## Conclusions

Surgical techniques for posterior stabilization of the subaxial spine include wiring, laminar screw fixation, lateral mass screw fixation, and pedicle screw fixation. Posterior cervical wiring is rarely performed today as a stand-alone procedure because it only offers resistance against flexion. Translaminar screw fixation is more frequently performed in the atlantoaxial and thoracolumbar spine, although it may be an option for subaxial spine fixation in selected cases when there are deficient lateral masses. Care must be taken to avoid laminar cortical breach and violation of the facet joint. Pedicle screws are the standard of care in the thoracic and lumbar spine but are technically challenging to place in the subaxial spine due to small pedicle diameter and high medial angulation in this region. Lateral mass screw fixation is the mainstay technique for posterior subaxial stabilization and offers exceptionally high fusion rates. Comprehensive preoperative planning is critical to evaluate for anatomical variations, and a sound knowledge of the anatomical relationships between screw trajectory and surrounding structures is key in avoiding and minimizing intraoperative and postoperative complications.

## References

[1] Hadra BE (1891). Wiring the spinous processes in Pott’s disease. Trans Am Orthop Assoc.

[2] Rogers WA (1942). Treatment of fracture-dislocation of the cervical spine. J Bone Joint Surg Am.

[3] Rogers WA (1957). Fractures and dislocations of the cervical spine: an end-result study. J Bone Joint Surg Am.

[4] Abdu WA, Bohlman HH (1992). Techniques of subaxial posterior cervical fusions: an overview. Orthopedics.

[5] Whitehill R, Stowers SF, Fechner RE, Ruch WW, Drucker S, Gibson LR, McKernan DJ, Widmeyer JH (1987). Posterior cervical fusions using cerclage wires, methylmethacrylate cement and autogenous bone graft. An experimental study of a canine model. Spine (Phila Pa 1976).

[6] Benzel EC, Kesterson L (1989). Posterior cervical interspinous compression wiring and fusion for mid to low cervical spinal injuries. J Neurosurg.

[7] Murphy MJ, Southwick WO (1989). Posterior approaches and fusions. The Cervical Spine, 2nd ed.

[8] Geremia GK, Kim KS, Cerullo L, Calenoff L (1985). Complications of sublaminar wiring. Surg Neurol.

[9] Watts C, Smith H, Knoller N (1993). Risks and cost-effectiveness of sublaminar wiring in posterior fusion of cervical spine trauma. Surg Neurol.

[10] Lovely TJ, Carl A (1995). Posterior cervical spine fusion with tension-band wiring. J Neurosurg.

[11] Vaccaro AR, Singh K (2003). Posterior wiring techniques of the spine. Surgical Techniques for the Spine.

[12] Whitehill R, Cicoria AD, Hooper WE, Maggio WW, Jane JA (1988). Posterior cervical reconstruction with methyl methacrylate cement and wire: a clinical review. J Neurosurg.

[13] McAfee PC, Bohlman HH, Wilson WL (1985). The triple wire fixation technique for stabilization of acute cervical fracture-dislocations: a biomechanical analysis. Trans Orthop.

[14] Omeis I, DeMattia JA, Hillard VH, Murali R, Das K (2015). History of instrumentation for stabilization of the subaxial cervical spine. Neurosurg Focus.

[15] Chiles BW, Cooper PR (2006). Trauma of the mid- and lower cervical spine. Atlas of Neurosurgical Techniques: Spine and Peripheral Nerves.

[16] Parker SL, McGirt MJ, Garcés-Ambrossi GL, Mehta VA, Sciubba DM, Witham TF, Gokaslan ZL, Wolinksy JP (2009). Translaminar versus pedicle screw fixation of C2: comparison of surgical morbidity and accuracy of 313 consecutive screws. Neurosurgery.

[17] Grob D, Humke T (1998). Translaminar screw fixation in the lumbar spine: technique, indications, results. Eur Spine J.

[18] Shin SI, Yeom JS, Kim HJ, Chang BS, Lee CK, Riew KD (2012). The feasibility of laminar screw placement in the subaxial spine: analysis using 215 three-dimensional computed tomography scans and simulation software. Spine J.

[19] Alvin MD, Abdullah KG, Steinmetz MP, Lubelski D, Nowacki AS, Benzel EC, Mroz TE (2012). Translaminar screw fixation in the subaxial cervical spine: quantitative laminar analysis and feasibility of unilateral and bilateral translaminar virtual screw placement. Spine (Phila Pa 1976).

[20] Hong JT, Sung JH, Son BC, Lee SW, Park CK (2008). Significance of laminar screw fixation in the subaxial cervical spine. Spine (Phila Pa 1976).

[21] Hong JT, Yi JS, Kim JT, Ji C, Ryu KS, Park CK (2010). Clinical and radiologic outcome of laminar screw at C2 and C7 for posterior instrumentation--review of 25 cases and comparison of C2 and C7 intralaminar screw fixation. World Neurosurg.

[22] Jang SH, Hong JT, Kim IS, Yeo IS, Son BC, Lee SW (2010). C7 posterior fixation using intralaminar screws : early clinical and radiographic outcome. J Korean Neurosurg Soc.

[23] Roy-Camille R, Saillant G, Mazel C (1989). Internal fixation of the unstable cervical spine by a posterior osteosynthesis with plates and screws. The Cervical Spine, 2nd ed.

[24] Nazarian SM, Louis RP (1991). Posterior internal fixation with screw plates in traumatic lesions of the cervical spine. Spine (Phila Pa 1976).

[25] Magerl F, Seemann PS (1987). Stable posterior fusion of the atlas and axis by transarticular screw fixation. Cervical Spine.

[26] Jeanneret B, Magerl F, Ward EH, Ward JC (1991). Posterior stabilization of the cervical spine with hook plates. Spine.

[27] Anderson PA, Henley MB, Grady MS, Montesano PX, Winn HR (1991). Posterior cervical arthrodesis with AO reconstruction plates and bone graft. Spine.

[28] Ebraheim N (1999). Posterior lateral mass screw fixation: anatomic and radiographic considerations. The University of Pennsylvania Orthopaedic Journal.

[29] Graham AW, Swank ML, Kinard RE, Lowery GL, Dials BE (1996). Posterior cervical arthrodesis and stabilization with a lateral mass plate. Clinical and computed tomographic evaluation of lateral mass screw placement and associated complications. Spine (Phila Pa 1976).

[30] Gill K, Paschal S, Corin J, Ashman R, Bucholz RW (1988). Posterior plating of the cervical spine: a biomechanical comparison of different posterior fusion techniques. Spine (Phila Pa 1976).

[31] Kast E, Mohr K, Richter HP, Börm W (2006). Complications of transpedicular screw fixation in the cervical spine. Eur Spin J.

[32] Pelton MA, Schwartz J, Singh K (2012). Subaxial cervical and cervicothoracic fixation techniques--indications, techniques, and outcomes. Orthop Clin North Am.

[33] Abumi K, Ito M, Sudo H (2012). Reconstruction of the subaxial cervical spine using pedicle screw instrumentation. Spine.

[34] Heller JG, Jeffords P (2004). Posterior instrumentation of the lower cervical spine. The Adult and Pediatric Spine, 3rd ed.

[35] Heller JG, Carlson GD, Abitbol JJ, Garfin SR (1991). Anatomic comparison of the Roy-Camille and Magerl techniques for screw placement in the lower cervical spine. Spine (Phila Pa 1976).

[36] O’Brien MF (2003). Surgical anatomy of the cervical spine. Spinal Deformities: The Comprehensive Text.

[37] Sekhon LH (2005). Posterior cervical lateral mass screw fixation: analysis of 1026 consecutive screws in 143 patients. J Spinal Disord Tech.

[38] Katonis P, Papadakis SA, Galanakos S, Paskou D, Bano A, Sapkas G, Hadjipavlou AG (2011). Lateral mass screw complications: analysis of 1662 screws. J Spinal Disord Tech.

[39] Rao R, Marawar SV (2013). Posterior cervica fusion with instrumentation. Operative Techniques in Spine Surgery.

[40] Sanelli PC, Tong S, Gonzalez RG, Eskey CJ (2002). Normal variation of vertebral artery on CT angiography and its implications for diagnosis of acquired pathology. J Comput Assist Tomogr.

[41] Karaikovic EE, Daubs MD, Madsen RW, Gaines RW Jr (1997). Morphologic characteristics of human cervical pedicles. Spine.

[42] Miller RM, Ebraheim NA, Xu R, Yeasting RA (1996). Anatomic consideration of transpedicular screw placement in the cervical spine. An analysis of two approaches. Spine.

[43] Karaikovic EE, Yingsakmongkol W, Gaines RW Jr (2001). Accuracy of cervical pedicle screw placement using the funnel technique. Spine.

[44] Ludwig SC, Kramer DL, Balderston RA, Vaccaro AR, Foley KF, Albert TJ (2000). Placement of pedicle screws in the human cadaveric cervical spine: comparative accuracy of three techniques. Spine.

[45] Kast E, Mohr K, Richter HP, Börm W (2006). Complications of transpedicular screw fixation in the cervical spine. Eur Spine J.

[46] Pelton MA, Schwartz J, Singh K (2012). Subaxial cervical and cervicothoracic fixation techniques--indications, techniques, and outcomes. Orthop Clin North Am.

[47] Abumi K, Ito M, Sudo H (2012). Reconstruction of the subaxial cervical spine using pedicle screw instrumentation. Spine.

[48] Johnston TL, Karaikovic EE, Lautenschlager EP, Marcu D (2006). Cervical pedicle screws vs. lateral mass screws: uniplanar fatigue analysis and residual pullout strengths. Spine J.

[49] Jones EL, Heller JG, Silcox DH, Hutton WC (1997). Cervical pedicle screws versus lateral mass screws. Anatomic feasibility and biomechanical comparison. Spine.

[50] Kothe R, Rüther W, Schneider E, Linke B (2004). Biomechanical analysis of transpedicular screw fixation in the subaxial cervical spine. Spine.

[51] Johnston TL, Karaikovic EE, Lautenschlager EP, Marcu D (2006). Cervical pedicle screws vs. lateral mass screws: uniplanar fatigue analysis and residual pullout strengths. Spine J.

[52] Abumi K, Ito M, Kaneda K (2000). Surgical treatment of cervical destructive spondyloarthropathy (DSA). Spine.

[53] Oda I, Abumi K, Ito M, Kotani Y, Oya T, Hasegawa K, Minami A (2006). Palliative spinal reconstruction using cervical pedicle screws for metastatic lesions of the spine: a retrospective analysis of 32 cases. Spine.

[54] Abumi K, Itoh H, Taneichi H, Kaneda K (1994). Transpedicular screw fixation for traumatic lesions of the middle and lower cervical spine: description of the techniques and preliminary report. J Spinal Disord.

[55] Hong JT, Tomoyuki T, Udayakumar R, Espinoza Orías AA, Inoue N, An HS (2011). Biomechanical comparison of three different types of C7 fixation techniques. Spine.

[56] Abumi K, Shono Y, Taneichi H, Ito M, Kaneda K (1999). Correction of cervical kyphosis using pedicle screw fixation systems. Spine.

[57] Abumi K, Takada T, Shono Y, Kaneda K, Fujiya M (1999). Posterior occipitocervical reconstruction using cervical pedicle screws and plate-rod systems. Spine.

[58] Abumi K, Kaneda K (1997). Pedicle screw fixation for nontraumatic lesions of the cervical spine. Spine.

[59] Abumi K, Shono Y, Ito M, Taneichi H, Kotani Y, Kaneda K (2000). Complications of pedicle screw fixation in reconstructive surgery of the cervical spine. Spine.

[60] Yoshimoto H, Sato S, Hyakumachi T, Yanagibashi Y, Masuda T (2005). Spinal reconstruction using a cervical pedicle screw system. Clin Orthop Relat Res.

[61] Yukawa Y, Kato F, Ito K, Horie Y, Hida T, Nakashima H, Machino M (2009). Placement and complications of cervical pedicle screws in 144 cervical trauma patients using pedicle axis view techniques by fluoroscope. Eur Spine J.

[62] Neo M, Sakamoto T, Fujibayashi S, Nakamura T (2005). The clinical risk of vertebral artery injury from cervical pedicle screws inserted in degenerative vertebrae. Spine.

[63] Tomasino A, Parikh K, Koller H, Zink W, Tsiouris AJ, Steinberger J, Härtl R (2010). The vertebral artery and the cervical pedicle: morphometric analysis of a critical neighborhood. J Neurosurg Spine.

[64] Pech P, Daniels DL, Williams AL, Haughton VM (1985). The cervical neural foramina: correlation of microtomy and CT anatomy. Radiology.

[65] Xu R, Kang A, Ebraheim NA, Yeasting RA (1999). Anatomic relation between the cervical pedicle and the adjacent neural structures. Spine.

[66] Richter M, Cakir B, Schmidt R (2005). Cervical pedicle screws: conventional versus computer-assisted placement of cannulated screws. Spine (Phila Pa 1976).

[67] Kotani Y, Abumi K, Ito M, Minami A (2003). Improved accuracy of computer-assisted cervical pedicle screw insertion. J Neurosurg.

[68] Kotil K, Akçetin MA, Savas Y (2012). Neurovascular complications of cervical pedicle screw fixation. J Clin Neurosci.

